# Colonic Leishmaniasis Followed by Liver Transplantation

**DOI:** 10.4269/ajtmh.2010.09-0430

**Published:** 2010-08-05

**Authors:** Stanley Almeida Araujo, Thaís Costa Nascentes Queiroz, Monica Maria Demas Alvares Cabral

**Affiliations:** Program of Infectious Diseases and Tropical Medicine, Faculty of Medicine, Federal University of Minas Gerais, Brazil; Pediatric Gastroenterology, Alfa Institute of Gastroenterology, Hospital of the Clinics, Federal University of Minas Gerais, Brazil; Department of Pathology, Alfa Institute of Gastroenterology, Hospital of the Clinics, Federal University of Minas Gerais, Brazil

**Figure 1.**
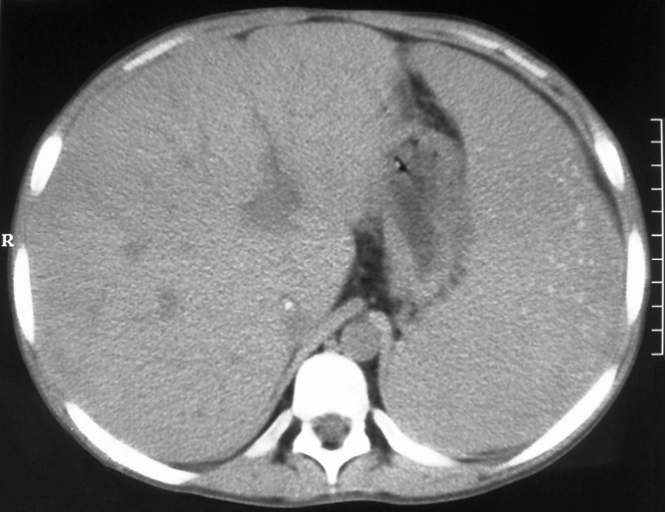
Computerized tomography of the abdomen showing hepatosplenomegaly.

**Figure 2.**
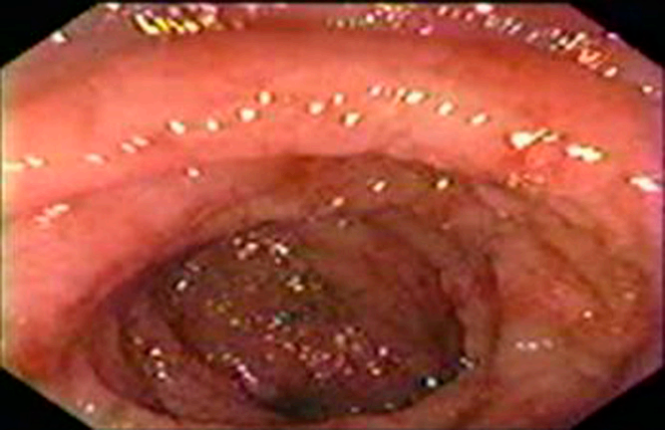
Colonoscopic view—irregular nodularity and hyperemia of the mucosa. This figure appears in color at www.ajtmh.org.

**Figure 3.**
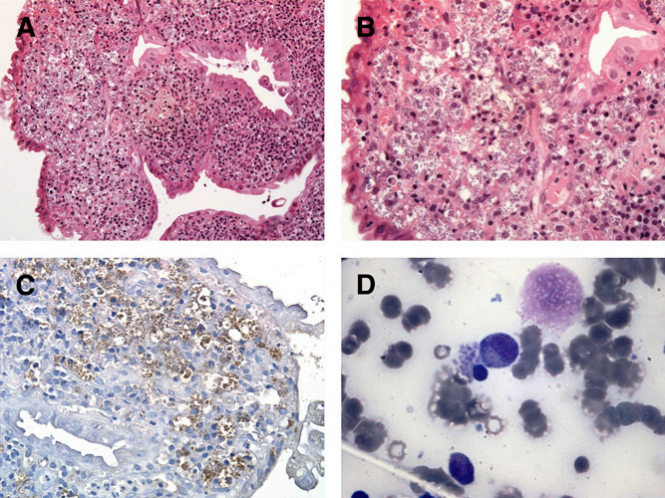
Biopsy of colon and bone marrow. (**A**) Increased cellularity of the colon because of mononuclear inflammatory infiltrate. (**B**) Detail shows macrophages filled with amastigote forms of *Leishmania chagasi*. (**C**) Positive immunohistochemical reaction by the streptavidin-biotin method, with specific staining of the amastigotes. (**D**) Amastigote-parasitized macrophage in bone-marrow aspirate. This figure appears in color at www.ajtmh.org.

A 17-year-old, 37-kg Brazilian man was diagnosed with autoimmune hepatitis at the age of 7 years and underwent liver transplant (cadaveric) at the age of 14. Immunosuppressants included tacrolimus and prednisone. He had lost 3 kg over the previous year and had diarrhea with blood streaks in the previous 3 months but no history of fever. Laboratory tests showed pancytopenia (hemoglobin = 8.1 g/dL; leukocytes = 1,300/mm^3^; platelets = 79,000/mm^3^; albumin = 2.7 g/dL). Physical examination showed enlarged liver and spleen. Unrevealing imaging included upper gastroenterointestinal endoscopy, abdominal ultrasound, and computerized tomography of the chest and abdomen (Figure 1) but showed hepatosplenomegaly. Viral serologies were negative. Colonoscopy showed gross nodularity, hyperemia, and friability of the colonic mucosa (Figure 2). Biopsy of terminal ileum region showed amastigote forms of *Leishmania* inside macrophages (Figure 3A and B). The nature of the agent was confirmed by immunohistochemistry (Figure 3C) and polymerase chain reaction (PCR), which showed *L. chagasi* infection.^1^ Examination of a bone-marrow biopsy showed innumerable *Leishmania* amastigotes (Figure 3D). Serology for leishmaniasis was negative. Treatment with amphotericin B desoxycholate was initiated; renal function deteriorated, and treatment with liposomal amphotericin was substituted (3 mg/kg for 7 days).^2^ The patient clinically responded with resolution of diarrhea, weight gain, and normalization of spleen size. Two months later, hemoglobin was 11.3 g/dL, leukocytes were 3,670/mm^3^, platelets were 164,000/mm^3^, and albumin was 3.8 g/dL.^1^,^2^
